# Pre-operative micronutrient deficiencies in patients with severe obesity candidates for bariatric surgery

**DOI:** 10.1007/s40618-020-01439-7

**Published:** 2020-10-07

**Authors:** M. Pellegrini, F. Rahimi, S. Boschetti, A. Devecchi, A. De Francesco, M. V. Mancino, M. Toppino, M. Morino, G. Fanni, V. Ponzo, E. Marzola, G. Abbate Daga, F. Broglio, E. Ghigo, S. Bo

**Affiliations:** 1 Department of Medical Sciences, University of Turin, c.so AM Dogliotti 14, 10126 Torino, Italy; 2 Unit of Clinical Nutrition, “Città della Salute e della Scienza” Hospital of Turin, Turin, Italy; 3 Department of Surgical Sciences, University of Turin, Turin, Italy; 4 Department of Neuroscience, University of Turin, Turin, Italy; 5 Diabetes and Metabolic Diseases Clinic, “Città della Salute e della Scienza” Hospital of Turin, Turin, Italy

**Keywords:** Bariatric surgery, C-reactive protein, Micronutrient deficiency, Severe obesity, Vitamin D

## Abstract

**Purpose:**

In patients with obesity, micronutrient deficiencies have been reported both before and after bariatric surgery (BS). Obesity is a chronic pro-inflammatory status, and inflammation increases the risk of micronutrient malnutrition. Our objective was to assess in pre-BS patients the prevalence of micronutrient deficiencies and their correlation with blood values of C-reactive protein (CRP).

**Methods:**

Anthropometric data, instrumental examinations, and blood variables were centrally measured in the first 200 patients undergoing a pre-BS evaluation at the “Città della Salute e della Scienza” Hospital of Torino, starting from January 2018.

**Results:**

At least one micronutrient deficiency was present in 85.5% of pre-BS patients. Vitamin D deficiency was the most prevalent (74.5%), followed by folate (33.5%), iron (32%), calcium (13%), vitamin B12 (10%), and albumin (5.5%) deficiency. CRP values were high (> 5 mg/L) in 65% of the patients. These individuals showed increased rate of iron, folate, vitamin B12 deficiency, and a higher number of micronutrient deficiencies. In a multiple logistic regression model, increased CRP levels were significantly associated with deficiencies of vitamin B12 (OR = 5.84; 95% CI 1.25–27.2; *p* = 0.024), folate (OR = 4.02; 1.87–8.66; *p* < 0.001), and with the presence of ≥ 2 micronutrient deficiencies (OR = 2.31; 1.21–4.42; *p* = 0.01).

**Conclusions:**

Micronutrient deficiencies are common in patients with severe obesity undergoing BS, especially when inflammation is present. In the presence of increased CRP values before surgery, it might be advisable to search for possible multiple micronutrient deficiencies.

## Introduction

The term malnutrition refers to undernutrition—including stunting (low height for age), wasting (low weight for height), underweight (low weight for age) and micronutrient deficiencies or insufficiencies (vitamin and mineral deficiencies)—and overweight/obesity [1]. The global nutritional transition with increased use of high-energy density and processed foods, calorie-rich seasonings, and sugar-sweetened beverages is contributing to the world obesity pandemic [2, 3]. An unbalanced nutrient intake with insufficient consumption of unrefined cereals, legumes, vegetables, and fruits may be responsible for the double burden of malnutrition with the coexistence in the same individuals of both obesity and multiple micronutrient deficiencies [4]. This seems especially true for people with severe obesity [5]. Paradoxically, the patients who are candidates to bariatric surgery are at risk for nutrient deficiencies not only postoperatively [5], but also before surgery [6]. Deficiencies of vitamin D [7–27], iron [7, 11–13, 15–18, 20, 21, 23–27], vitamin B12 [9, 12–14, 17, 18, 21, 23, 24, 26, 27], folate [13, 16–18, 22, 25–28], and zinc [9, 13, 14, 29] are the most frequently reported. In a few studies, deficiencies of pyridoxine [11, 12, 22], magnesium [11, 15], thiamine [7], copper [13], vitamin A [30], and vitamin K [18] have been described.

Increased BMI seems to be directly associated with the risk of micronutrient deficiencies [9, 13, 15, 18, 20, 24–28]. Younger patients are more likely to show nutritional deficiencies than older [7, 15, 18, 24, 26–28], female sex is significantly associated with iron deficiency [7, 9, 17–20, 23–25, 27, 28], but various gender differences were found depending on the specific nutrient deficiency [9, 10, 19, 20, 23, 24, 26].

It is well known that undernutrition and inflammation are strictly inter-related, inflammation being an important factor increasing the risk for both undernutrition and its adverse health effects [31]. Among the acute phase reactants whose blood concentrations increase during inflammation, C-reactive protein (CRP) is one of the most important and it is now considered a disease marker in many chronic non-communicable diseases, with clinical, pathological, and therapeutic significance [32]. Patients with obesity and increased visceral adipose tissue show a chronic pro-inflammatory status [33, 34]. No studies have currently evaluated the possible association between inflammation and micronutrient malnutrition in patients with severe obesity.

Therefore, this paper aimed to assess the micronutrient deficiencies and their possible association with increased blood levels of high-sensitivity CRP in patients with severe obesity seeking bariatric surgery (BS).

## Methods

This was a cross-sectional study. We collected the data of the first 200 patients who underwent a pre-operative evaluation at the “Città della Salute e della Scienza” Hospital of Torino, starting from January 2018 and meeting the following inclusion criteria: (1) age 18–65 years; (2) BMI ≥ 35 kg/m^2^ with comorbidities or BMI ≥ 40 kg/m^2^, with numerous unsuccessful attempts to lose weight; (3) candidates to BS (either sleeve gastrectomy or Roux-en-Y gastric bypass).

Exclusion criteria were the following: (1) secondary causes of obesity (e.g. hypothalamic diseases, endocrine diseases); (2) patients with acute inflammatory conditions; (3) associated comorbidities or diseases contraindicating BS; (4) previous BS.

### Pre-operative management

All patients underwent the following assessments:

-Clinical examination by surgeons, anesthetists, psychiatrists, endocrinologists, dieticians, and MDs with specific expertise in the management of patients undergoing BS.
-measurement of weight, height, waist and neck circumference, and arterial blood pressure.
-Dietary history and assessment of habitual daily food intake and eating habits, taken by a trained registered dietitian.
-Collection of a fasting blood sample for the measurement of high-sensitivity CRP, glucose, glycated hemoglobin (HbA1c), total and HDL-cholesterol, triglycerides, creatinine, uric acid, γ-glutamil transferase (GGT), hemoglobin, iron, transferrin, ferritin, albumin, vitamin B12, 25(OH) vitamin D, folate, calcium, phosphate, magnesium, sodium, potassium.
-Electrocardiogram.
-Arterial blood gas analysis.
-Chest X-ray.
-Abdomen ultrasound.
-Esophagogastroduodenoscopy.
-Further exams, when necessary (e.g. polysomnography, echocardiogram, visits by cardiologist, pulmonologist, gastroenterologist, neurologist with sleep expertise, etc.).

Pre-BS, all patients received verbal and written dietary, exercise, and behavioral recommendations. An individually prescribed diet was given in accordance with the Mediterranean diet composition (45–55% carbohydrates, < 10% sugars, 30% fats, < 10% saturated fats, 15–25% proteins, 20–30 g fiber) with an energy restriction ranging from 500 to 1000 kcal and based on the individual caloric requirements and usual intake.

### Measurements

Weight was measured with patient wearing light clothes and no shoes to the nearest 0.1 kg by a digital scale with 300 kg capacity (Wunder Sa.Bi.srl). Height was measured to the nearest 0.1 cm with a Stadiometer SECA 220 measuring rod (Hamburg, Germany). Waist and neck circumferences were assessed by a plastic tape meter at the umbilicus level and under the cricoid cartilage, respectively.

Arterial blood pressure values were measured from the left arm, in a sitting position, after at least 10 min of rest, with a mercury sphygmomanometer and appropriate cuff sizes (ERKA Perfect-Aneroid, Germany).

### Laboratory methods

Fasting blood glucose, albumin, iron, inorganic phosphate, calcium, magnesium, triacylglycerol, HDL-cholesterol, total cholesterol, uric acid, GGT, creatinine, sodium, potassium, high-sensitivity CPR, and transferrin were determined on serum by immunometric assays on the Roche Cobas 800 modular analyzer (Roche Diagnostic GmbH, Mannheim, Germany).

Ferritin, folic acid, vitamin B12, and 25(OH) vitamin D were determined on serum by immuno-enzymatic assays on the Roche Cobas 800 modular analyzer (Roche Diagnostic GmbH, Mannheim, Germany). Hemoglobin and mean cellular volume (MCV) were determined on the Sysmex XN-module for hematology automated analyzer (Sysmex Co., Kobe, Japan). Glycated hemoglobin (HbA1c) was determined on whole blood using an ion-exchange high-performance liquid chromatography (HPLC) on the D-100 Hemoglobin Testing System (BioRad Laboratories, Redmond, WA, USA).

### Diagnosis

Super-obesity was defined in the presence of a BMI ≥ 50 kg/m^2^. Type 2 diabetes mellitus (T2DM) was diagnosed by a MD in accordance with guidelines [35]. The diagnosis of non-alcoholic fatty liver disease (NAFLD) was made in the presence of hepatic steatosis at the ultrasound examination, in the absence of other causes for secondary hepatic fat accumulation; hiatal hernia/gastroesophageal reflux was detected by esophagogastroduodenoscopy. Obstructive sleep apnea (OSA) was hypothesized in the presence of excessive daytime sleepiness, snoring, and choking or gasping during sleep, enlarged neck circumference, and an intermediate to high-risk score at the STOP-Bang questionnaire [36]. The diagnosis was confirmed by a sleep-expert neurologist by means of further exams (in-laboratory polysomnography, unattended home sleep apnea testing, etc.). The diagnosis of osteoarthritis was self-reported.

Corrected serum calcium (mg/dL) was calculated by using the following formula and then transformed in mmol/L: {measured serum calcium (mg/dL) + [0.8 × (4.0—albumin (g/dL)]}.

Reference ranges for CRP were selected according to the reference range of our laboratory that are based on the published international Consensus reference ranges [37].

The following deficiencies were considered, according to our laboratory reference ranges:

-Iron [transferrin saturation < 15% (females), < 20% (males)].
-Albumin levels < 3.6 g/dL.
-Vitamin B12 levels < 200 pg/mL.
-25(OH) Vitamin D levels < 20 ng/mL.
-Folate levels < 3.0 ng/mL.
-Calcium corrected for albumin < 2.12 mmol/L.
-Phosphate levels < 0.81 mmol/L.
-Magnesium levels < 0.62 mmol/L.
-Sodium levels < 135 mmol/L.
-Potassium levels < 3.5 mmol/L.

Anemia was defined in the presence of hemoglobin values < 12 g/dL in females and < 13.5 g/dL in males.

After the results of the laboratory tests, patients underwent nutritional counseling and were prescribed specific nutritional supplementation.

### Statistical analyses

Patients were divided according to their CRP values ≤ 5 or > 5 mg/L. Between-group differences were analyzed by *t* Student test and ANOVA, or Wilcoxon Rank and Kruskal–Wallis test, as appropriate. The Chi-squared test was used to compare dichotomous variables. Pairwise Spearman’s non-parametric correlations were used to evaluate the relationships between CRP levels and nutrient values.

A logistic regression analysis was performed to evaluate the variables significantly associated with nutritional deficiencies (dependent variables), after adjustments for age, sex, BMI, and glycated hemoglobin. The relative contribution of direct and indirect effects was assessed through mediation analysis.

## Results

Most patients were women, with many obesity-related complications, above all NAFLD; 13.5% showed BMI ≥ 50 kg/m^2^ (Table 1). Among patients with T2DM, 22 were on metformin, 6 on glucagon-like-peptide-1 agonists, 5 on gliflozins, 5 on insulin, and 1 on dipeptidyl-peptidase-4-inhibitors. None of the participants was taking supplements before laboratory analyses. Mean habitual total energy intake was 2598.8 ± 471.2 kcal, with fats = 36.2 ± 4.0% total kcal, carbohydrates = 47.5 ± 5.6% total kcal, and proteins = 16.3 ± 3.8% total kcal (0.94 ± 0.26 g/kg). The vast majority of participants (86%) were inactive or had a low level of exercise.

**Table 1:** Characteristics of the patients

	Mean (or percentage)	SD
Age (years)	43.2	10.9
Males (%)	21.5	
Weight (kg)	114.7	21.6
Height (m)	1.63	0.92
BMI (kg/m2)	42.8	6.6
Super-obesity (BMI ≥ 50 kg/m^2^) (%)	13.5	
Waist circumference (cm)	125.2	16.1
Neck circumference (cm)	41.1	4.3
Systolic blood pressure (mmHg)	133.7	10.7
Diastolic blood pressure (mmHg)	81.5	4.6
Active smoker (%)	29.5	
Diabetes mellitus (%)	19.0	
Arterial blood hypertension (%)	34.0	
Non-alcoholic fatty liver disease (%)	88.0	
Obstructive Sleep Apnea (%)	20.0	
Osteoarthritis (%)	41.0	
Hiatal hernia/gastroesophageal reflux disease (%)	47.5	

Most of participants (85.5%) had at least one micronutrient deficiency, about a half showed 2 or more deficiencies. There was a high prevalence of vitamin D deficiency, although mostly mild (53%, with levels 10–20 ng/mL; 21.5% with levels < 10 ng/mL). Iron deficiency (as suggested by a low % transferrin saturation) was higher, though not significantly different, in females (34.4% vs. 23.3%, *p* = 0.17) (Table 2). The prevalence of anemia was indeed low (11.5%; 12.1% in females and 9.3% in males, *p* = 0.61). Among patients with anemia, 82.6% had low iron levels, 43.5% folate deficiency, 17.4% low vitamin B12, 34.8% combined deficiencies, and 8.7% thalassemia trait. A few individuals (*n* = 11) showed low albumin levels. No deficiency for sodium, potassium, magnesium was found; 13% of individuals had low calcium (corrected for albumin) levels and only three low phosphate levels.

**Table 2:** Laboratory values of the patients

	Mean or percentage	SD, (interquartile range)
CRP (mg/L)	7.00^a^	(9.5)
Fasting glucose (mg/dL)	98.2	35.4
Glycated hemoglobin (%)	6.0	1.2
Total cholesterol (mg/dL)	196.0	35.5
HDL cholesterol (mg/dL)	50.5	12.7
Triglycerides (mg/dL)	144.5	68.8
Creatinine (mg/dL)	0.76	0.19
Uric acid (mg/dL)	5.5	1.4
γ-glutamil transferase (IU/L)	22.5^a^	(18.5)
Hemoglobin (g/dL)	13.7	1.4
Anemia (%)^b^	11.5	
Mean cell volume (fL)	85.5	6.7
Iron (µg/dL)	80.3	40.2
Transferrin (mg/dL)	283.7	61.5
Ferritin (ng/mL)	73.5^a^	(111.5)
Transferrin saturation (%)	19.7^a^	(11.8)
Iron deficiency (%)^c^	32.0	
Albumin (g/dL)	4.3	0.38
Low albumin (< 3.6 g/dL) (%)	5.5	
Vitamin B12 (pg/mL)	403.8	160.8
Vitamin B12 deficiency (< 200 pg/mL) (%)	10.0	
25 (OH) Vitamin D (ng/mL)	16.4	7.5
25 (OH) Vitamin D deficiency (< 20 ng/mL) (%)	74.5	
Folate (ng/mL)	3.9^a^	(3.0)
Folate deficiency (< 3.0 ng/mL) (%)	33.5	
Calcium (mmol/L)	2.3	0.12
Calcium corrected for albumin (mmol/L)	2.2	0.12
Low calcium corrected for albumin (< 2.12 mmol/L) (%)	13.0	
Phosphate (mmol/L)	1.0	0.18
Low phosphate (< 0.81 mmol/L) (%)	1.5	
Magnesium (mmol/L)	0.81	0.06
Sodium (mEq/L)	140.4	2.5
Potassium (mEq/L)	4.4	0.41
Number of micronutrient deficiencies (%) 0	15.5	
1	36.5	
≥ 2	48.0	

^a^Median values

^b^Hemoglobin values < 12 g/dL (females), < 13.5 g/dL (males)

^c^Transferrin saturation < 15% (females), < 20% (males)

Active smokers showed a significantly higher prevalence of folate deficiency (49.2% vs. 27.0%, *p* = 0.002). Individuals with super-obesity showed more frequently anemia (25.9% vs. 9.3%, *p* = 0.011) and iron deficiency (59.3% vs. 27.8%, *p* = 0.001). Patients with OSA showed more frequently vitamin D deficiency (87.5% vs. 71.3%, *p* = 0.035). Metformin users had a higher, though not significantly different, prevalence of vitamin B12 deficiency than non-users (18.2% vs. 9.0%, *p* = 0.18).

High CRP values were present in 65% of the patients (Table 3). Individuals with higher CRP levels showed higher fasting glucose and HbA1c values, with a higher proportion of females. Median CRP values were significantly higher in patients with T2DM (9.5 vs. 6.5 mg/L, *p* = 0.043) and super-obesity (12.0 vs. 6.6 mg/L, *p* = 0.023).

**Table 3:** Characteristics of the patients by CRP values

	CRP ≤ 5 mg/L	CRP > 5 mg/L	*p*
Number	70	130	
Age (year)	44.9 ± 10.4	42.2 ± 11.1	0.09
Males (%)	30.0	16.9	**0.032**
Weight (kg)	114.9 ± 20.7	114.5 ± 22.1	0.90
BMI (kg/m2)	42.5 ± 5.9	43.0 ± 6.9	0.61
Super-obesity (BMI ≥ 50 kg/m^2^)	12.9	13.9	0.85
Waist circumference (cm)	123.8 ± 15.7	126.0 ± 16.3	0.36
Neck circumference (cm)	41.4 ± 4.9	40.9 ± 4.0	0.37
Systolic blood pressure (mmHg)	134.1 ± 10.1	133.4 ± 11.1	0.68
Diastolic blood pressure (mmHg)	81.5 ± 3.8	81.4 ± 5.0	0.91
Active smoker (%)	24.3	32.3	0.06
Diabetes mellitus (%)	12.9	22.3	0.10
Metformin treatment (%)	10.0	11.5	0.74
Arterial blood hypertension (%)	30.0	36.2	0.38
Non-alcoholic fatty liver disease (%)	88.6	87.7	0.86
Obstructive Sleep Apnea (%)	27.1	16.2	0.06
Osteoarthritis (%)	32.9	45.4	0.09
Hiatal hernia/gastroesophageal reflux disease (%)	40.0	51.5	0.12
Fasting glucose (mg/dL)	91.1 ± 14.7	102.1 ± 42.1	**0.035**
Glycated hemoglobin (%)	5.7 ± 0.70	6.2 ± 1.38	**0.010**
Total cholesterol (mg/dL)	189.8 ± 33.8	199.4 ± 36.1	0.07
HDL cholesterol (mg/dL)	51.1 ± 11.6	50.2 ± 13.3	0.65
Triglycerides (mg/dL)	135.8 ± 72.9	149.2 ± 66.4	0.19
Creatinine (mg/dL)	0.79 ± 0.20	0.75 ± 0.18	0.09
Uric acid (mg/dL)	5.7 ± 1.4	5.4 ± 1.4	0.30
γ-glutamil transferase (IU/L)^a^	21.0 (16.0)	23.0 (19.0)	0.10
Hemoglobin (g/dL)	13.8 ± 1.5	13.6 ± 1.4	0.58
Anemia (%)^b^	10.0	12.3	0.63
Mean cell volume (fL)	85.8 ± 8.11	85.3 ± 5.8	0.65
Iron (µg/dL)	91.9 ± 53.7	74.0 ± 29.0	**0.003**
Transferrin (mg/dL)	276.8 ± 56.3	287.3 ± 64.0	0.25
Ferritin (ng/mL)^a^	75.0 (134.0)	68.0 (109.0)	0.73
Transferrin saturation (%)^a^	22.0 (9.6)	18.0 (9.3)	**0.002**
Iron deficiency (%)^c^	22.9	36.9	**0.041**
Albumin (g/dL)	4.4 ± 0.30	4.2 ± 0.41	**0.003**
Low albumin (< 3.6 g/dL)	1.4	7.7	0.06
Vitamin B12 (pg/mL)	425.6 ± 154.2	392.1 ± 163.7	0.16
Vitamin B12 deficiency (< 200 pg/mL) (%)	2.9	13.9	**0.013**
25 (OH) Vitamin D (ng/mL)	16.8 ± 7.1	16.2 ± 7.8	0.59
25 (OH) Vitamin D deficiency (< 20 ng/mL) (%)	71.4	76.2	0.46
Folate (ng/mL)^a^	4.9 (2.6)	3.3 (2.8)	** < 0.001**
Folate deficiency (< 3.0 ng/mL) (%)	15.7	43.1	** < 0.001**
Calcium (mmol/L)	2.29 ± 0.11	2.29 ± 0.12	0.97
Calcium corrected for albumin (mmol/L)	2.25 ± 0.13	2.22 ± 0.11	**0.049**
Low calcium corrected for albumin (< 2.12 mmol/L) (%)	11.5	15.7	0.40
Phosphate (mmol/L)	1.0 ± 0.19	1.0 ± 0.18	0.71
Low phosphate (< 0.81 mmol/L) (%)	0	2.3	0.20
Magnesium (mmol/L)	0.81 ± 0.07	0.80 ± 0.06	0.27
Sodium (mEq/L)	141.8 ± 2.1	141.1 ± 2.7	0.06
Potassium (mEq/L)	4.3 ± 0.42	4.4 ± 0.41	0.39
Number of micronutrient deficiencies (%) 0	22.9	11.5	
1	44.3	32.3	
≥ 2	32.9	56.2	**0.005**

^a^Mann–Whitney test

^b^Hemoglobin values < 12 g/dL (females), < 13.5 g/dL (males)

^c^Transferrin saturation < 15% (females), < 20% (males)

Statistically significant values are in bold (*p* < 0.05)

Patients with increased CRP levels displayed lower levels of percentage transferrin saturation, albumin, folate, and calcium corrected for albumin. Deficiencies of iron, folate, vitamin B12, and the number of micronutrient deficiencies were significantly higher in individuals with increased CPR levels. Median CRP values increased with the number of micronutrient deficiencies (4.8, 6.2, 9.0 mg/L, *p* = 0.002 by Kruskal–Wallis test). Inverse relationships were evident between CRP levels and albumin (Rho = − 0.37, *p* < 0.001), iron (Rho = − 0.28; *p* < 0.001), percentage transferrin saturation (Rho = − 0.29, *p* < 0.001), vitamin B12 (Rho = − 0.19, *p* < 0.006), folate (Rho = − 0.33, *p* < 0.001), and vitamin D (Rho = − 0.16, *p* = 0.027).

In a multiple logistic regression model, increased CRP levels were significantly associated with deficiencies of folate and vitamin B12 and the presence of ≥ 2 micronutrient deficiencies (Table 4). The results of the logistic regression model did not change significantly after adjusting for waist circumference, smoking habits, metformin use, presence of OSA, and super-obesity. The observed effect sizes (i.e., the observed ORs) were similar to those found at univariate analysis, empirically suggesting the absence of important mediation effects by the covariates considered in the adjusted model (Table 4). This result was specifically confirmed by mediation analysis, in which no statistically significant indirect effects mediated by age, sex, BMI, and glycated hemoglobin were found.

**Table 4:** Association between micronutrient deficiencies and increased CRP values in logistic regression models and at mediation analysis

	Univariate regression			Multivariate regression^a^			Indirect effects°
	OR	95% CI	*p*	OR	95% CI	*p*	*p*
Iron deficiency^**b**^	1.98	1.02–3.85	**0.044**	1.78	0.88–3.60	0.11	0.23
Vitamin B12 deficiency^**c**^	5.46	1.22–24.5	**0.026**	5.84	1.25–27.2	**0.024**	0.94
Folate deficiency^**d**^	4.06	1.94–8.47	** < 0.001**	4.02	1.87–8.66	** < 0.001**	0.45
≥ 2 nutritional deficiencies	2.62	1.42–4.82	**0.002**	2.31	1.21–4.42	**0.010**	0.12

^**a**^Model adjusted for age, sex, BMI, glycated hemoglobin. Each row is a model

^**b**^Transferrin saturation < 15% (females), < 20% (males)

^**c**^Vitamin B12 < 200 pg/mL

^**d**^Folate < 3.0 ng/mL

°*p* values for the presence of indirect effects mediated by age, sex, BMI, glycated hemoglobin at mediation analysis

## Discussion

About a half of adults with severe obesity candidates for BS showed ≥ 2 micronutrient deficiencies, being by far vitamin D deficiency the most common. This was the first study evaluating the possible association between the inflammatory status and micronutrient deficiencies. We found that increased CRP values were strongly associated with vitamin B12 and folate deficiencies and the presence of multiple micronutrient deficiencies.

Long-term micronutrient deficiencies after BS are frequent as a consequence of either non-compliance to recommendations or altered digestion and decreased absorption because of the BS-induced anatomic changes, sustained post-operative nausea, and vomiting, food intolerance, and gut bacterial overgrowth consuming fat-soluble and B1/B12 vitamins [5, 38]. Indeed, multiple micronutrient deficiencies have been reported even before BS, leading to the need for specific dietary recommendations and supplement prescription for most of those patients [5, 38]. We have found that 15% of the pre-BS patients only showed no micronutrient deficiency. Other studies have shown the presence of nutritional deficiencies in a highly variable percentage of pre-BS patients [5, 6, 38]. Differences in age, gender distribution, ethnicity, previous treatment and/or supplementation, cutoffs and criteria for deficiency definition, geographical and seasonal factors might explain discrepancies among studies. None of our patients was taking supplements, differently from other studies with high proportions of individuals on supplements [10, 19]. Nevertheless, most studies consistently found micronutrient deficiencies in > 50% of the pre-BS patients [5, 38].

Individuals with obesity are at increased risk of multiple micronutrient deficiencies for their poor diet quality, because of incorrect dietary regimens to lose weight, abnormal storage, and bioavailability of nutrients and reduced sun exposure (for vitamin D) [5, 38]. Indeed, a high frequency (around or higher 50%) of lower than recommended intakes of iron, calcium, folic acid, and magnesium was found despite a high caloric intake [11, 19, 21, 29], thus suggesting a poor-quality diet lacking in micronutrients. A chronic pro-inflammatory state is frequent in patients with obesity [8, 14]. Accordingly, 65% of our patients showed increased CRP levels.

Inflammation might both aggravate and contribute by itself to undernutrition [31] by increasing hepcidin synthesis leading to reduced iron absorption [38], inducing the depletion of essential amino-acids, such as tryptophan [39], and the deficiencies of some vitamins, including vitamin B6 [40]. In contrast, an inflammatory state might be the consequence of micronutrient malnutrition: vitamin D deficiency is associated with a high prevalence of inflammation and vitamin D supplementation slightly reduced CRP levels in individuals with obesity [41]. Many molecular mechanisms have been implicated in the anti-inflammatory effects of this vitamin [42]. Similarly, folic acid supplementation significantly lowered CRP serum levels, probably owing to the improvement of insulin resistance, oxidative stress, and hyperhomocysteinemia [43]. Finally, many foods and their bioactive components, such as vegetables, fruits, extra-virgin olive oil, red wine, nuts, and specific food pattern, such as the Mediterranean diet, might exert anti-inflammatory effects and the opposite happens with a western-type diet [44]. Inflammation and micronutrient malnutrition are, therefore, strongly associated, and it is difficult to disentangle the causal role of one another. We found an increased risk for micronutrient malnutrition in the presence of higher CRP values, after adjustment for several possible confounders. One study analyzed the associations between CRP levels and micronutrient deficiencies before BS and found inverse relationships between CRP and blood magnesium, zinc, folate, albumin, and hemoglobin [14].

Hypoalbuminemia was relatively common after BS (up to 18%) [38], but quite rare before surgery in most studies (0–6%) [7, 11, 13–16, 28, 45], except a few [9, 23, 26]. We found it in 5.5% of our patients. Indeed, the chronic low-grade inflammatory state of obesity might have reduced the levels of acute phase proteins, such as albumin; therefore, the observed hypoalbuminemia should be carefully interpreted, since it may not necessarily indicate a deficiency state.

Vitamin D deficiency was by far the most frequent deficiency, consistently with literature [7–11, 13, 15–18, 20–27]; poor diet, the limited bioavailability of vitamin D, its increased uptake by the adipose tissue, and a reduced sunlight exposure might justify the high prevalence of this deficiency. Its potential role in the pathogenesis of obesity and dysmetabolic and cardiovascular diseases is yet to be defined [46, 47]. Furthermore, it has been reported that systemic inflammation could impact vitamin D serum levels [48]. We did not find significant differences in vitamin D levels by CRP values; the complexity of the interrelationships between obesity, inflammation, and vitamin D levels could have obscured possible associations; otherwise, other factors could be more important in determining its circulating levels in the presence of abundant adipose tissue [46, 47]. Intriguingly, patients with OSA showed more frequently low vitamin D blood levels in accordance with literature [49]. The vitamin D deficiency was incrementally exacerbated with increasing OSA severity [49, 50]. Increased BMI and larger neck circumference, systemic inflammation, hypoxia-induced mechanisms, excessive daytime sleepiness with reduced outdoor activities, and genetic variations increasing the susceptibility to both conditions have been considered to explain this association [49–51].

Folate deficiency varied from 0% [10, 23] to 63% [25] among pre-BS patients; most studies found folate deficiency rates between 15 and 30% [13, 16–18, 22, 26–28]. Folate is not stored in the body, and a diet lacking in this nutrient (i.e., poor in green leafy vegetables, citrus fruits, lentils, beans, and fortified grains) can lead to folate depletion in few weeks [5]. Furthermore, the human gut microbiota (in particular, some strains of *Lactobacillus* and *Bifidobacterium*) is known to produce folate, being the human microbiome potentially capable of synthesizing 37% of the daily recommended intake of folate for non-pregnant, non-breastfeeding adults [52, 53], and impairments in the microbiota composition and function have been repeatedly described in obesity [54]. Dysbiosis and inflammation are strongly related; the fourfold higher risk of folate deficiency of our individuals with increased CRP values is in line with the scientific evidence and suggests complex relationships between the nutritional status and the entire human ecosystem.

In pre-bariatric patients, the deficiency of vitamin B12 ranges from 0% [19] to 34.4% [24], being 5–15% [12, 13, 15–17, 21, 22, 25–27] the most frequently reported prevalence. The high prevalence of gastroesophageal reflux disease, use of proton pump inhibitors and metformin, both reducing vitamin B12 absorption, might justify this deficiency. The human gut microbiota produces vitamin B12 and the obesity-related dysbiosis might contribute both to altered vitamin production and increased pro-inflammatory state [55]. Accordingly, the risk of vitamin B12 deficiency was more than fivefold higher in our patients with increased CRP values, and the association remained after adjusting for metformin use.

Prevalence of anemia in our patients was low, in line with literature [8, 9, 16, 17, 27, 45] and mostly due to iron deficiency. The latter has been reported with a frequency between 6% [45] and 59% [23], also due to the different definitions, based either on blood levels of iron, ferritin, or percentage of transferrin saturation. Our females and individuals with super-obesity showed higher iron deficiency rate, underlining the importance of lower iron storing capacity in females, iron loss with menses, and dilutional factors [5]. An impaired iron absorption due to increased hepcidin synthesis contributed to explain the iron deficiency in individuals with obesity and the associations with inflammation [56]. In our patients, the relationships between iron deficiency and CRP levels were no longer significant in the multiple regression model. Iron deficiency was estimated as a low percentage of transferrin saturation, which was calculated as the ratio of iron to transferrin; both iron and transferrin are acute phase reactants, thus potentially interacting with many factors and not directly representing the nutritional status.

### Clinical considerations

Managing pre-BS patients is an unresolved challenge. A few studies have evaluated nutritional deficits before and after surgery, showing either similar prevalence [57], mixed results, i.e., improvement in some nutrients and worsening in others [16, 17, 22, 29] or, more frequently, exacerbation [8, 12, 30, 45, 58–61]. In particular, when abstracting patients with pre-bariatric micronutrient deficiency, sleeve gastrectomy itself only had a modest effect on post-BS micronutrient status [12, 17]. Furthermore, the presence of preoperative deficiencies was the strongest predictor of their presence in the postoperative period [12, 17, 30]; micronutrient malnutrition has been linked to adverse surgical outcomes [7–9, 11, 17, 27, 62, 63] and surgical complications may interfere with tolerance to nutritional supplementation postoperatively, being adherence to supplementation much easier before surgery [12]. Finally, few intervention studies have demonstrated that identification and treatment of the micronutrient deficiencies preoperatively can prevent their worsening and ameliorate outcomes postoperatively [64, 65].

At present, there is a low level of evidence regarding the micronutrients to be followed pre-operatively, the screening markers for nutritional deficiencies, the definitions of pathological cutoffs, or the doses for nutritional supplements preoperatively [66]. Routine comprehensive micronutrient panels are controversial and costly, and the clinical relevance of these deficiencies and their treatment algorithms need to be better defined. In consideration of the increased frequency of multiple nutritional deficiencies in inflamed patients, the possibility to subject them to a more extensive testing than recommended by guidelines [66] before undergoing BS might be considered. Further larger intervention trials should be performed in order to assess whether preoperative vitamin and micronutrient supplementation to correct their deficiency may impact on the outcomes of surgery in those individuals.

In conclusion, increased levels of CRP (a simple and low-cost measurement) might be a potential marker of micronutrient deficiencies pre-BS; intriguingly, post-BS CRP changes are increasingly demonstrated to predict weight regain and unfavorable surgery outcomes, thus suggesting a relevant prognostic role for this marker in relation to BS [67, 68].

### Strengths and limitation

The possible association between the inflammatory status and micronutrient deficiencies in patients with severe obesity had never been studied before. This was a real-life study with centralized blood measurements, and standardization of all the procedures.

We analyzed neither red blood cell folate levels (which can be normal in individual with obesity) nor other micronutrients such as zinc, selenium, copper, and other vitamins. The evaluated micronutrients were those usually measured in the clinical practice and whose deficiencies were the most commonly observed in our patients after BS. We did not evaluate the micronutrient intake of our patients; indeed their energy and/or macronutrient intakes were in line with previous studies reporting a high-calorie diet poor in micronutrients in pre-BS patients [11, 19, 21, 60].

The cross-sectional design of the study prevented conclusions about causality to be drawn. The majority of our patients were women; indeed, the same gender distribution was reported by most published studies as well. Finally, the sample size was small, but the post-hoc power analysis found that the study had an 89% power (with α = 0.05) to detect differences between the CRP groups.

## Conclusion

Micronutrient deficiencies are common in patients undergoing BS, especially when they are inflamed. In the presence of increased CRP values before surgery, the search for possible micronutrient deficiencies might be advisable. Further studies are needed to understand whether pre-bariatric nutritional supplementation might have an impact on surgical outcomes and reduce the risk of post-bariatric nutritional deterioration.
